# Serendipitous identification of *Pratylenchus curvicauda* from the grainbelt of Western Australia

**DOI:** 10.21307/jofnem-2019-046

**Published:** 2019-07-29

**Authors:** Farhana Begum, John Fosu-Nyarko, Shashi Sharma, Bill Macleod, Sarah Collins, Michael G. K. Jones

**Affiliations:** 1 Plant Biotechnology Research Group, WA State Agricultural Biotechnology Centre, College of Science, Health, Engineering and Education, Murdoch University, Perth, WA, 6150, Australia; 2 Department of Primary Industries and Regional Development, South Perth, WA, 6151, Australia

**Keywords:** Internal transcribed spacer (ITS), Molecular phylogeny, Morphology, Nematode classification, *Pratylenchus* spp, *Pratylenchus curvicauda*, Root Lesion Nematodes, Scanning Electron Microscopy (SEM), Taxonomy

## Abstract

A *Pratylenchus* species identified during a survey of *Pratylenchus quasitereoides* incidence at four locations of the grainbelt of Western Australia is described. Morphological and morphometric features indicated the previously undescribed morphotypes in nematode mixtures encountered were conspecific to *P. curvicauda*, and were clearly distinguishable from nine common *Pratylenchus* spp. Typical features of *P. curvicauda* were its body length (415–540 µm), which was curved to a c-shaped with a maximum body diameter of 20 µm, and the nature of its tail; 34 µm long, 2.8 µm wide at the anus and a typical ventrally arcuate with a round terminus. Sequenced for the first time, the sequences of the partial 18S-ITS1-5.8S-ITS2-partial 28S (80 clones, 14 individual nematodes) and the 28S-D3 (17 clones) regions of the rDNA of *P. curvicauda* had overall mean distances of 0.013 and 0.085, respectively. Phylogenetic analyses with sequences of both segments of the rDNA clearly showed the *P. curvicauda* isolates as monophyletic, distinct from ca 40 *Pratylenchus* species. Notably, it was distinct from *Pratylenchus* species present in Australia including *P. quasitereoides* and a Western Australia isolate of *P. thornei*. Further research into the biology of *P. curvicauda* is needed to facilitate development of strategies for its management, if it is an important pest.

Root lesion nematodes (*Pratylenchus* spp.) are one of three economically important plant-parasitic nematode pests of many crops worldwide (Jones et al., 2013). The over 77 species currently described are polyphagous and cause yield losses of major grain crops including wheat and barley and even more significant damage under drought conditions (Taylor et al., 1999; Castillo and Vovlas, 2007). In Australia, about 12 economically important *Pratylenchus* species have been described and these include the recently described *P. quasitereoides* (Hodda et al., 2014) (previously *P. teres* Khan and Singh, 1974) (Hodda and Nobbs, 2008; Jones and Fosu-Nyarko, 2014). They are often present as mixed populations in infested soils. Seven of these species are known to be present in the southern and western grainbelts of Australia. These are *P. neglectus* (Rensch, 1924; Filipjev and Stekhoven, 1941), *P. thornei* (Sher and Allen, 1953), *P. quasitereoides*, *P. penetrans* (Cobb, 1917; Filipjev and Stekhoven, 1941), *P. zeae* (Graham, 1951), *P. brachyurus* (Godfrey, 1929; Filipjev and Stekhoven, 1941), and *P. scribneri* Steiner in Sherbakoff and Stanley (1943). Whereas *P. neglectus* is the most common in this region, *P. thornei* is the main pest of wheat and barley in the northern grainbelt of the continent (Vanstone et al., 2008). A recent survey on the distribution of *Pratylenchus* species in 360 paddocks in Western Australia indicated *P. neglectus* was the most prevalent (48% incidence) with 32% of the paddocks surveyed estimated to have mixed infestations, usually with *P. quasitereoides* (Collins et al., 2017). Knowledge of plant-parasitic nematode species present in any infested field is essential because their management could be undermined by a shift to a predominance of species for which crops grown are not resistant (Jones and Fosu-Nyarko, 2014). This shift emphasizes the need for accurate identification of the species present in an infested field for successful management of infestations.

Since the discovery and first description of *P. curvicauda* in metropolitan Perth, Western Australia, in 1991 (Siddiqi et al., 1991), there has been no further study on the nematode in Australia. As such, its pest status and existence in the grainbelt of Australia is not known, and no specific management strategy is in place for this potential pest of wheat, barley, and other important crops. There is extensive ongoing research on the identification and management of root lesion nematodes in Western Australia, but none currently includes *P. curvicauda* (Collins et al., 2015, 2017). One possible reason is the overlapping morphological and morphometric features of root lesion nematodes that often make it difficult to distinguish between species accurately (Castillo and Vovlas, 2007). Hence, molecular approaches and phylogenetic analyses tools have been combined to distinguish species, as together, they offer greater accuracy and reproducibility. These tools are also adaptable for nematode diagnostics, as long as the original specimens used as standards were identified correctly using traditional methods (Al-Banna et al., 1997; Subbotin et al., 2008; De Luca et al., 2011). As for many other organisms, the genes encoding the ribosomal RNA subunits have proved to be useful in taxonomic studies of nematodes. These genomic regions vary in their rate of evolution depending on whether they encode functional products or not. They include those of the ribosomal small subunit genes which can be extremely conserved, or the non-coding internal transcribed spacer (ITS) regions which are much more variable between species of the same genera (Mckeand, 1998). Sequencing of the ITS regions has revealed species-specific variations, which can be used as diagnostic markers, so enabling accurate identification of species and studies on the phylogenetic relationships between and within species of *Pratylenchus* (Waeyenberge et al., 2009; Palomares-Rius et al., 2010). Also, the nucleotide sequences of the D2 to D3 regions of the large subunit ribosomal genes (28S), which is thought to evolve slowly, have been used to examine the evolutionary relationships among species of many genera including *Pratylenchus* (Al-Banna et al., 1997).

*Pratylenchus teres*, which was previously considered to be endemic to Western Australia, has recently been re-described as *Pratylenchus quasitereoides* using traditional methods and sequences of the 28S-D3 region of the rDNA (Hodda et al., 2014). The latter species is reported to occur with *P. neglectus* in Katanning, Western Australia. In a recent survey to study the prevalence of *P. quasitereoides* in four wheat and barley fields of the grainbelt of Western Australia, initial assessment of the morphometrics of isolated nematodes indicated the features of some nematodes did not conform to those of *Pratylenchus* spp. commonly reported. This study was, therefore, undertaken to describe the species which was prominent in the mixed population of *Pratylenchus* species found: we report the use of morphometric measurements, morphological features, and genetic variation within the partial 18S-ITS1-5.8S-ITS2-partial 28S and 28S-D3 regions of the rDNA to characterise *P. curvicauda* and to distinguish it from other *Pratylenchus* species including those commonly found in Australia. This exercise is an important step in assessing the pest status and economic importance of the nematode to the grains industry in Western Australia.

## Materials and methods

### Nematode population

**Figure 1:**
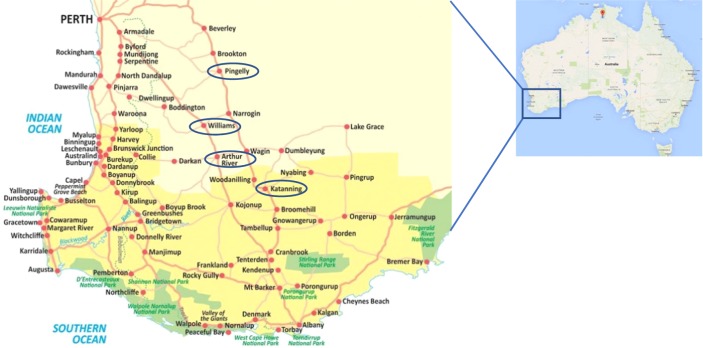
Locations of the four wheat/barley paddocks in the Western Australian grainbelt where *P. curvicauda* samples were isolated. The four sites are circled; Pingelly, Williams, Arthur River, and Katanning (https://maps-australia.com/south-western-australia-map).

Soil samples containing a mixture of root lesion nematodes including *P. curvicauda* were obtained from four different locations in the wheatbelt region of Western Australia: Pingelly (32°32′2.4″S, 117°5′9.6″E), Williams (33°2′0″S, 116°53′0″E), Arthur River (33°20′19″S, 117°2′4″E), and Katanning (33°41′27″S, 117°33′19″E), with the help of staff at the Plant Pathology Section, Nematology Division of the Department of Primary Industries and Regional Development, Western Australia (Fig. 1). Nematodes were extracted from the soils using a misting apparatus described by Tan et al. (2013). A pure culture of *P. thornei* maintained on carrot disks at 23°C in our laboratory at the time of this experiment was used as a control during the morphological characterization. The partial 18S-ITS1-5.8S-ITS2-partial 28S and 28S-D3 expansion sequences of the *P. thornei* were also sequenced for the first time and used to differentiate the *P. curvicauda* reported in this study.

### Morphological studies and morphometric measurements

Initial identification of the *P. curvicauda* was carried out using two important morphological features: the position of the vulva and the shape of the tail. The morphology of individual specimens was examined and photographed using a compound microscope (Olympus BX51). Before the examination, single nematodes were hand-picked using a fine feather and placed in a drop of water on a glass slide. The slide with nematode was quickly passed over a flame of a Bunsen burner to stop the nematode from moving. Morphological measurements which included nematode body length and the position of the vulva from the tail were measured from captured images using the scale bars of the image software package on the Olympus BX51 compound microscope. Based on the latter measurements, the percent distance of the vulva from the anterior end of the nematode body, *V*, was calculated. Comparative morphometric and light microscopy images of immobilized *P. curvicauda* from the four experimental locations and those of *P. thornei* were also conducted using a Zeiss Axioskop Upright microscope (Carl Zeiss Microscopy, LLC, USA) at the Center for Microscopy, Characterization and Analysis, University of Western Australia, Perth. Nematode specimens from Pingelly with typical *P. curvicauda* features were fixed in 4% formaldehyde solution and sent to an expert taxonomist in the UK for further characterization. The detailed morphometric measurements obtained were compared with those taken in Australia.

### Preparation of nematode samples for scanning electron microscopy

Scanning electron microscopy (SEM) was used to further characterize *P. curvicauda.* To do this, single nematodes were fixed in 3% glutaraldehyde in 0.025 M phosphate buffer (pH 7.0) overnight at 4°C followed by five washes in the same buffer. The specimens were then fixed with 1% osmium tetroxide (OsO_4_) in 0.025 M phosphate buffer (pH 7.0) for 2 hr at room temperature in a fume hood, followed by five washes in the same buffer. The samples were dehydrated in a graded ethanol series (30%, 50%, 70%, and 90%), twice in each solution, for 15 min at a time. The 90% ethanol was then removed and replaced with 100% ethanol and then amyl acetate following the same regime. Specimens were then dried in a critical point dryer (FL-9496 BALZERS, Furstentum Liechtenstein). The nematodes were transferred to an SEM holder with conductive carbon tape and coated with a combination of 3 nm platinum and 10 nm carbon. The samples were then examined and images were taken using an SEM (Zeiss Ultra 55) at 5 KV.

### DNA extraction from single nematodes

Genomic DNA from individual adult female nematodes from the soil mixtures with typical *P. curvicauda* features and from pure cultures of *P. thornei* was extracted for PCRs using a modified protocol employing a worm lysis buffer (Wood, 1988). Each nematode was transferred onto a microscope slide and 10 μL of sterile water added. The nematode was then carefully diced with a fine scalpel blade after which the fragments were transferred into 50 μL of lysis solution (1% SDS, 50 mM EDTA, 100 mM NaCl, 100 μg/ml proteinase K, 1% 2-mercaptoethanol, 100 mM Tris-HCl pH 8.5) in a 1.5 mL centrifuge tube. The nematode lysate was then frozen at −80°C for 40 min followed by thawing to room temperature and then heating at 60°C for a further 40 min. The suspension was then centrifuged at 1,000 g for 2 min and the supernatant transferred to a fresh tube for extraction of nucleic acids using phenol-chloroform (PC:50:50). The PC-lysate emulsion was vortexed for a minute and centrifuged at 16,000 g for 2 min. The supernatant was added to one-tenth volume of 3 M NaOAc (pH 6.8) and 2.5 volumes of 100% ethanol, stored at −80°C overnight before being centrifuged at 16,000 g for 30 min to Analyses of sequenc pellet DNA. The pellet was washed twice with 400 μL of 70% ice-cold ethanol, dried in a fume hood and resuspended in 17 μL of nuclease-free water. The DNA was quantified using a spectrophotometer (Nanodrop ND-1000, Isogen Life Sciences) and stored at −20°C until use.

### PCR, cloning, and sequencing

The DNA sequence of the partial 18S-ITS1-5.8S-ITS2-partial 28 S of the rDNA and part of the 28S-D3 expansion region were used to characterize and distinguish between *P. curvicauda* and other *Pratylenchus* species. The primer pair 18S-Int (5′-CGTAACAAGGTAGCTGTAGG-3′) and 26S-Int (5′-CCTCCGCTAAATGATATGC-3′) (De Luca et al., 2011) was used to amplify the partial 18S-ITS1-5.8S-ITS2-partial 28S region using the GoTaq^®^ Green Master Mix (Promega Corporation, Australia), a premixed ready-to-use solution containing 50 units/mL Taq DNA polymerase, 400 μM each of dATP, dGTP, dCTP, and dTTP with 3 mM MgCl_2_ and 1 to 2 μL of the genomic DNA. The reactions were incubated at 95°C for 5 min; 35 cycles of 95°C for 30 sec, 55°C for 5 sec, and 72°C for 1.40 min followed by a final step of 72°C for 10 min. The 28S-D3 region of the rDNA was amplified with the primer pair D2-F (5′-GACCCGTCTTGAAACACGGA-3′) and D3-R (5′-TCGGAAGGAACCAGCTACTA-3′) (De Luca et al., 2004) using the same PCR reagents but with the following temperature profile: 94°C for 6 min, followed by 35 cycles of 94°C for 1 min, 55°C for 1 min, 72°C for 1 min, and a final step of 72°C for 6 min. PCR products were observed on 1% agarose gel stained with SYBR Safe (Invitrogen Pty Ltd, Australia). The amplicons were cut out of the gel with clean sterile blades and the DNA purified using the Wizard^®^ SV Gel and PCR Clean-Up System (Promega Corporation, Australia). The DNA was sequenced using Sanger sequencing and cloned using the pGEM-T Easy vector system following the manufacturers’ protocol (Promega Corporation, Australia). Plasmid DNA was isolated using the Wizard^®^ Plus SV Minipreps DNA Purification System (Promega Corporation, Australia). Both strands of six randomly selected clones of amplicons from individual nematodes were sequenced using the Big Dye 3.1 dye terminator in an AB 3730 96 capillary DNA Sequencer (Applied Biosystems, Australia).

### Analyses of sequenced rDNA and phylogenetic relationships

Sequence profiles of the clones of amplicons of the partial 18S-ITS1-5.8S-ITS2-partial and 28S-D3 expansion regions of *P. curvicauda* and *P. thornei* were edited using Geneious (V8.1.8) (Kearse et al., 2012). Consensus sequences were then made after alignment with both CLUSTAL O (1.2.3) (Mcwilliam et al., 2013) and Geneious. Computations in further sequence analyses were reduced by using representative/consensus sequences of the clones from nematodes isolated from the different locations. Phylogenetic relationships of the *P. curvicauda* with other *Pratylenchus* spp. were constructed using the generated rDNA sequences of *P. curvicauda* and *P. thornei* and those of similar regions of other *Pratylenchus* species retrieved from the National Center for Biotechnology Information (NCBI) databases using both keyword searches and the BLASTn tool (https://blast.ncbi.nlm.nih.gov/Blast.cgi). Phylogenetic analyses were done with MEGA7 (Tamura et al., 2013), using all the different parameters such as substitution models, rates and patterns, treatments of gaps/missing data, and tree inference methods employed by the Maximum Likelihood, the Neighbor-Joining and the Minimum-evolution approaches to determine the most consistent tree representing the relationship of the *P. curvicauda* samples with other *Pratylenchus* species. These trees were constructed using the bootstrap method as a test of Phylogeny with 1,000 replicates, where necessary they are presented with the bootstrap values. Representative sequences of *Meloidogyne* spp. and *Radopholus similis,* derived from similar regions of the rDNA, were used as outgroups for the phylogenetic analyses.

Differences between and within sequences of either the partial 18S-ITS1-5.8S-ITS2-partial 28S or the 28S-D3 regions of isolates of the *Pratylenchus* species studied were estimated using overall mean distances. The overall mean distance is an estimate of evolutionary divergence between any group of sequences and is a measure of the number of base substitutions per site between sequences compared. All such analyses were conducted using the Maximum Composite Likelihood model using MEGA7 where codon positions included were 1st+2nd+3rd+non-coding and all positions containing gaps and missing data were eliminated (Tamura et al., 2004; Kumar et al., 2016).

Sequences of clones of the partial 18S-ITS1-5.8S-ITS2-partial 28S and the 28S-D3 regions of isolates of the *P. curvicauda* and *P. thornei* have been deposited in the nucleotide database of NCBI with the GenBank accession numbers MN010380-MN010412 and MN006333-MN006351, respectively. The accession numbers are also appended to the clones shown in Figures 6 and 7.

## Results

### Initial identification of *P. curvicauda* based on key morphological features

**Figure 2:**
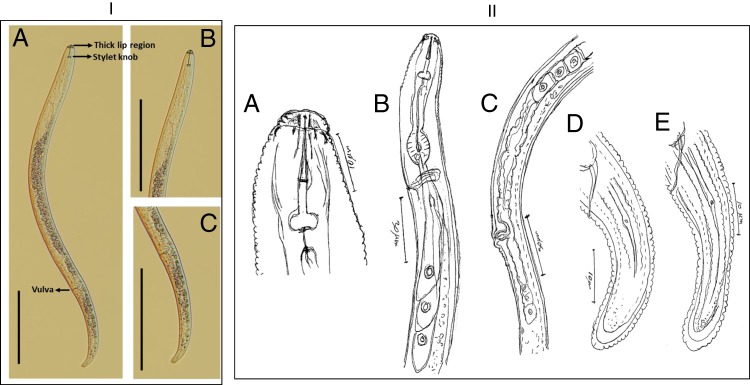
Light micrograph and line drawings of *P. curvicauda* from Pingelly, Western Australia [I] (A). Whole female adult nematode, (B) head region, (C) tail region (scale bar = 100 μm). [II]. Hand drawings of *P. curvicauda* from Pingelly, Western Australia: (A) head region, (B) Esophageal region, (C) vulva region, (D and E) tail region (Illustrations by the late Dr M. R. Siddiqi).

Over 90% of nematodes isolated from the soil collected from the wheat and barley paddocks at Pingelly, Arthur River, Katanning and Williams, and previously stored at 4°C, were plant-parasitic nematodes. Under a compound microscope, features observed in adult females included the characteristic stylet, thick lips, a stylet knob and overlapping esophageal regions that clearly distinguished them as root lesion nematodes (Fig. 2I). Three important morphological features, namely, the tail shape, the body length, and the vulva position, *V*, were further used to distinguish *P. curvicauda* from the mixture of nematodes in these soils. The identification exercise was aided with comparisons to pure cultures of Western Australian isolates of *P. thornei* maintained on carrot discs in our laboratory, and published data on other common species, namely, *P. neglectus, P. penetrans, P. quasitereoides, P. teres, and P. curvicauda*. Description of the nematodes studied are presented below.

### Morphological description of adult female *P. curvicauda*

Using the tail shape, body length and the *V*, *P. curvicauda* adult females were carefully isolated from nematode mixtures for further characterization. In total, 12 adult female nematodes from soil collected from Pingelly, 6 from Williams, 10 from Katanning, and 6 from Arthur River with the characteristic curvy tail were assessed further. The body length of all the *P. curvicauda* specimens varied from 441 μm to 768 μm with an average of 538 μm whereas the *V* ranged from 70 to 76, with an average of 74. The average body of adult female nematodes isolated from the Pingelly soil was 550 μm long (range 484-616 μm) with an average *V* of 74 (range 70-76). The respective average body lengths of the specimens from Williams, Arthur River, and Katanning soils were 520 μm (range 464-590 μm), 534 μm (range 478-636 μm) and 538 μm (range 441–768 μm) and the respective *V*s for these nematodes were 74 (range 71-76), 73 (70-76), and 74 (72-77).

The morphology of isolated nematodes from the Pingelly soil conspecific to *P. curvicauda* (*n* = 12) was described in detail in both laboratories, in Perth, and in the UK by the renowned nematologist, the late Dr M. R. Siddiqi. The descriptions are below.

### Head

**Figure 3:**
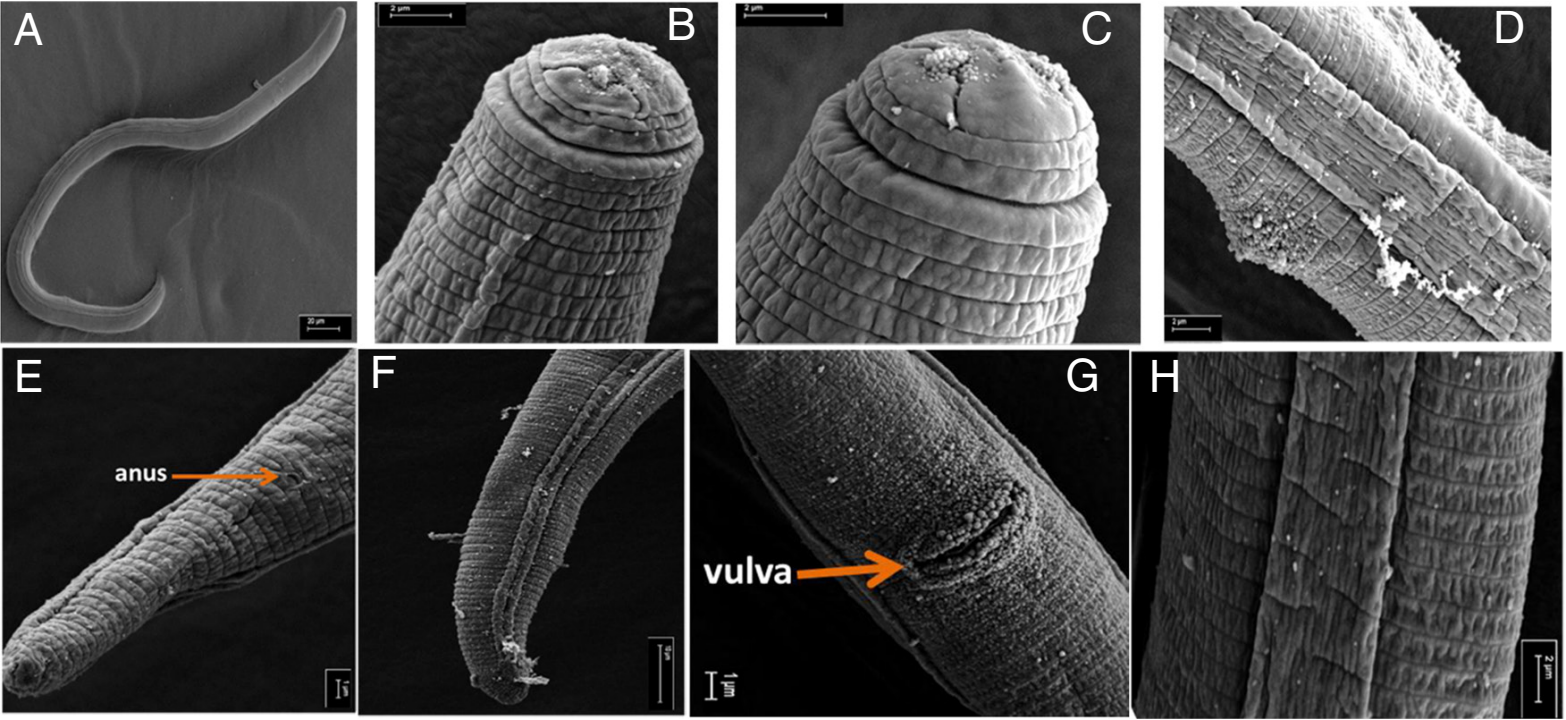
Scanning electron micrographs of an adult female *P. curvicauda* from Pingelly, Western Australia. (A) Whole nematode body, (B-C) *En face* view, (D) lateral field of the vulval region, (E) tail terminus. (F) tail region, (G) vulval region; (H) lateral field at middle of the body.

The *en face* of the head revealed a characteristic oral disc, which was slightly raised and divided (Fig. 3B,C). The head was rounded and was offset by a constriction, about 9 µm in diameter and 2.5 to 3 µm high with three distinct annules (Figs. 2IIa and 3IIB,C). The head framework was strongly sclerotised with its outer margins extending two annules into the body (Figs. 2IIa and 3IIB,C). These annules were set off from the body by a deep circular groove (cuticle constriction). Posterior to the circular constriction was an extremely wide first body annulus, which was evidently wider than the following body annules (Fig. 3B,C).

The spear was strong, with the conus being 51 to 55 percent of the spear length (Fig. 2IIa). The basal knobs were rounded, 5 µm across and 2 µm high. The orifice of the dorsal esophageal gland was at 3 to 4 µm posterior to the basal knobs (Fig. 2IIb). Two ventrosublateral esophageal glands were evident, ventral to the intestine, whereas the dorsal esophageal gland was anterior-most (Fig. 2IIa,b).

### Body

The body of the nematodes was typically curved to a c-shaped with a maximum diameter of 20 µm (Fig. 2I,II). The lateral fields had four incisures, the outer ones were distinctly crenate, with five to six incisures seen in the vulval region (Fig. 2IIc). Some of the adult females studied may have been gravid as an anterior ovary with a single row of oocytes was visible near the esophageal glands. No sperm was visible in the spermatheca. The post vulval uterine sac was differentiated in most of the nematodes, with a few reduced cells of the posterior ovary, 1.5 to 1.7 times the vulval body width long (Fig. 2IIc,d). The vulva was slightly protruding (Fig. 3A).

### Tail

The tail of the nematode was sub-cylindroid with the terminal fourth usually appearing lozenge-shaped, ventrally arcuate, terminus-rounded, and occasionally with an indentation (Figs. 2IId,e, 3E,F). The lateral field of the tail had four incisures in the anterior third, then with three incisures as it narrows down to the end just before the terminus. The phasmid was dot-like, usually with seven to nine annules or one-fourth of the tail length behind the anal level (Fig. 2IId,e).

### Male

No male nematode was identified from mixtures collected from any of the four locations, which is consistent with a *Pratylenchus* life cycle.

### Morphometric features of adult female *P. curvicauda*

Seven detailed morphometric measurements of *P. curvicauda* (Pingelly) were taken and compared to similar published data for six *Pratylenchus* species including *P. curvicauda,* previously described in metropolitan Western Australia in 1991, *P. teres* and the recently re-described *P. quasitereoides* isolated from Katanning, Western Australia. Despite the overlapping morphometric features typical of *Pratylenchus* species, the ranges and averages of the parameters studied for the Pingelly samples were more similar to, and clearly indicated that the samples were conspecific to *P. curvicauda* (Table 1). The average body length of the *P. curvicauda* from Pingelly falls within the range reported for five *Pratylenchus* species except for the published *P. neglectus* which are generally shorter (Table 1). The *a* of the Pingelly specimens was similar to those of *P. neglectus, P. penetrans, P. curvicauda*, and *P. teres,* but depicts the species as significantly different from *P. quasitereoides* and *P. thornei* (Table 1). Similarly, despite the overlap in the range of *b*, the average values for the *P. curvicauda* species were similar to those for *P. neglectus, P. penetrans,* and *P. thornei* but could clearly distinguish it from *P. quasitereoides* and *P. teres* (Table 1). The average value of *b*′ for the Pingelly samples was similar to that of the published *P. curvicauda*, 3.37 and 3.4, respectively, and the other species except for *P. quasitereoides* where the range was outside of those for the other species and the average was almost twice as much for *P. curvicauda* from Pingelly. Whereas the *c′* and the *V* for all the species in Table 1 were in range, the relative smaller value of *c* of the *P. curvicauda* distinguishes it from the other five species (Table 1).

**Table 1.** Comparative morphometric measurements of *Pratylenchus curvicauda* from Pingelly, Western Australia with other *Pratylenchus* species.

	*P. curvicauda* (Pingelly, Western Australia)	*P. curvicauda* (Siddiqi et al., 1991)	*P. neglectus* (Mizukubo and Minagawa, 1991)	*P. penetrans* (Ryss, 1988)	*P. thornei* (Pourjam et al., 1999)	*P. quasitereoides* (Hodda et al., 2014)	*P. teres* (Carta et al., 2002)
Body length (μm)	484–616	450–550	390–440	410–700	420–680	569–741	500–640
*a*	20.7–27 (24)	21–28 (24)	19.8–26.0 (23.1)	19–30 (24)	23–37 (31)	14–25 (18)	20.0–29.8 (24)
*b*	6.0–6.5 (6.2)	5.2–6.8 (5.9)	5.0–6.2 (5.5)	5.3–6.7 (5.6)	5.2–8.0 (6.5)	6.3–8.7 (7.3)	3.7–4.9 (4.4)
*b’*	3.3–3.37 (3.37)	3–3.9 (3.4)	3.3–4.4 (3.8)	–	3.3–5.4 (4.4)	4.9–10.1 (7.0)	–
*c*	12.9–14.6 (14.0)	13–18 (14.5)	16.6–20.3 (18.1)	15–24 (23)	15–26 (20)	15.9–22.5 (18.8)	15.4–18.4 (16.8)
*c’*	2.4–3.0 (2.8)	2.1–2.9 (2.7)	2.0–2.5 (2.3)	1.5–2.5 (2.1)	1.9–3.5 (2.6)	2.0–3.1 (2.3)	1.9–2.6 (2.2)
*V*	70–76 (74)	69–76.5 (73)	80–82 (81)	77–83 (80)	72–82 (77)	75–82 (78)	71–77 (75)

Notes: a = body length/greatest body diameter; b' = body length/distance from anterior to esophago-intestinal valve; b = body length/distance from anterior to base of esophageal glands; c = body length/tail length; c' = tail length/tail diameter at anus or cloaca; V = % distance of vulva from anterior; values in parenthesis ( ) represent average values.

### Diagnostics and relationships

Generally, characters that distinguished *P. curvicauda* from other *Pratylenchus* species were: the longer conus which was more than half of the spear length, the three annules in the lip region, the large rounded basal knobs, and the presence of phasmids in the anterior region of the tail which were usually about one-fourth the tail length behind the anus.

**Figure 4:**
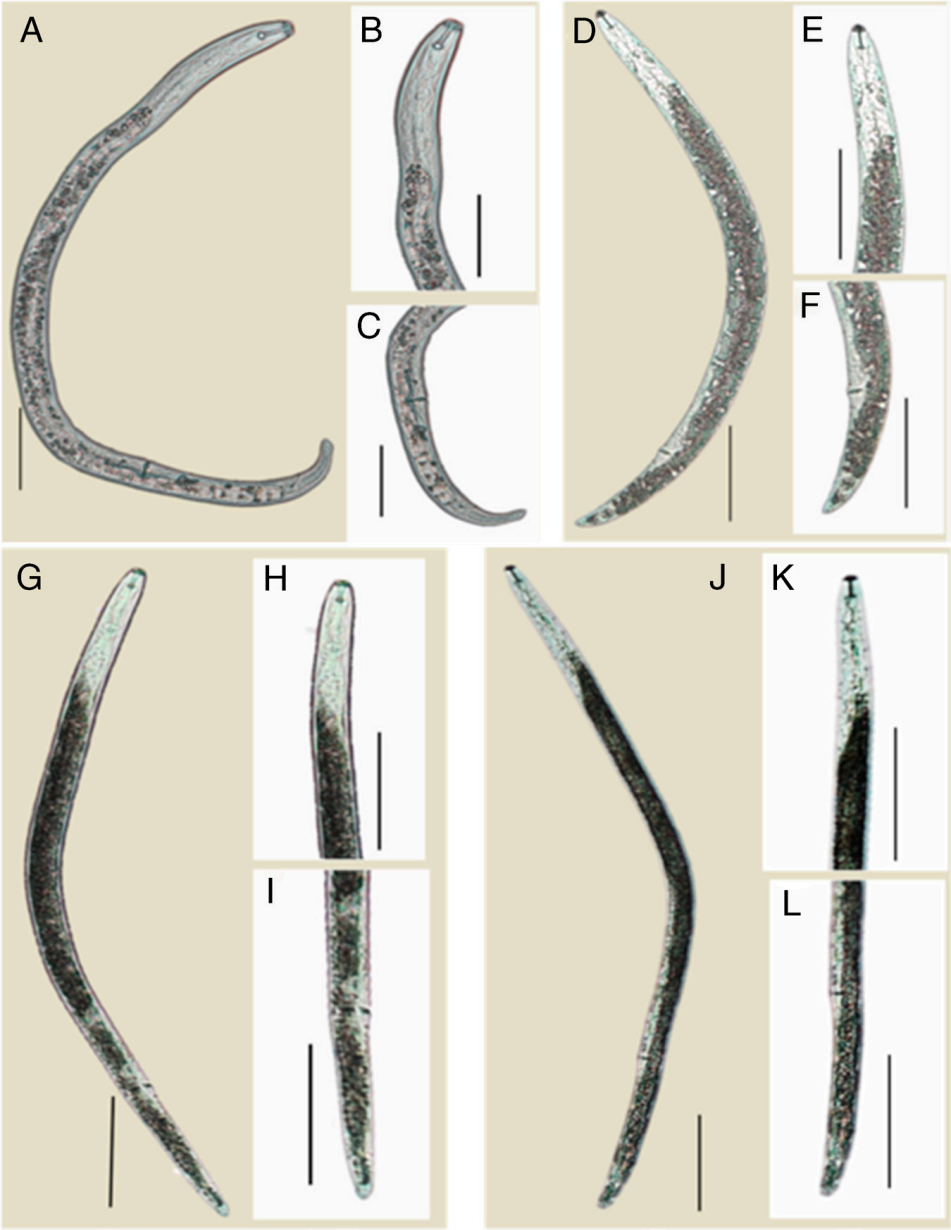
Light micrographs of the tail shapes of *P. curvicauda* and *P. thornei* (Western Australia) compared to nematode representative of *P. neglectus* and *P. penetrans* (A–C) *P. curvicauda* from Pingelly – (A) entire female body, (B) head region and (C) tail region (D–F) *P. neglectus*, (D) entire female body, (E) head region and (F) tail region (G–I) *P. penetrans*, (G) entire female body, (H) head region and (I) tail region (J–L) *P. thornei* (J) entire female body, (K) head region and (L) tail region (scale bar: A–C = 50 µm and D–L = 100 µm).

The body length and *V* was different from the Western Australia isolate of *P. thornei* cultured in our laboratory [*P. thornei*: *n* = 16, body length = 650 μm (range 557–809 μm), *V* = 76 (range 72–80)]. Also, the curved tail shape of *P. curvicauda* differed markedly from those of *P. neglectus* and *P. penetrans:* the tail of *P. penetrans* is generally rounded, and that of *P. neglectus* is conoid and for *P. thornei* the tail is conical with a round tip (Fig. 4).

Several features of *P. curvicauda* distinguish it from *P. quasitereoides.* First, the lateral field of *P. curvicauda* is wider and has more incisures in the vulval region. Also, for the stylet, the conus is longer than the shaft including the basal knobs, and this appears to be larger. The tail of *P. curvicauda* is more arcuate ventrally and its terminus is smooth except for occasional single indentation, while that of *P. quasitereoides* was depicted as having a broadly rounded, finely crenate terminus tail. In addition, the tail pattern of *P. curvicauda* is also quite different since the inner incisures go past the phasmids which are at the end of anterior fourth of the tail, while they are behind the middle of the tail for *P. quasitereoides.*

### ITS sequences of *P. curvicauda* and relationships with other *Pratylenchus* species

**Figure 5:**
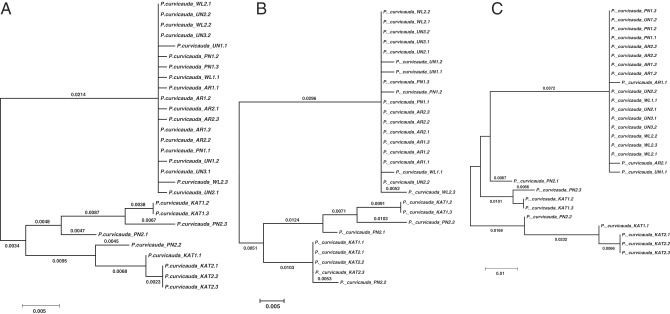
Molecular phylogenetic trees of representative sequences of the partial 18S-ITS1-5.8S-ITS2-partial 28S region of the rDNA of *P. curvicauda* isolates from soils of the grainbelt collected from four known (Arthur River, Pingelly, Williams, Katanning) and one unknown (UN) location in Western Australia. (A) Phylogenetic tree of sequences of the partial 18S-ITS1-5.8S-ITS2-partial regions, (B) Phylogenetic tree of sequences of ITS1 region, (C) Phylogenetic tree of sequences of the ITS2 region.

The partial 18S-ITS1-5.8S-ITS2-partial 28S sequences from six randomly selected clones for each amplicon from 14 adult females of *P. curvicauda* were analyzed. They included two nematodes each isolated from soils of Pingelly, Arthur River and Katanning, four from Williams and four from soil previously isolated from wheat and barley fields of unknown location in the grainbelt region of Western Australia. The nematodes from the unknown location included in this study were among a mixture of root lesion nematodes from the wheat field and also had typical features of *P. curvicauda*. The amplicons were cloned because direct sequencing yielded ambiguous peaks that indicated they were of mixed templates. For maximum quality assurance, both strands of plasmid DNA of each clone were sequenced. The sequences of the 24 clones of the nematodes from Williams, the 12 from clones of nematodes from Arthur River and those from the Unknown location (UN) were 906 to 907 nucleotides (nt) long whereas the 12 clones of the nematodes from both Katanning (928-933 nt) and Pingelly (906-933 nt) were generally longer. Based on sequence homology, the 84 clones were grouped into 28 unique sequences which included six clones each from the nematodes isolated from Pingelly (PN), Arthur River (AR), Katanning (KAT) and the Unknown location with four for the 24 clones from the four nematodes in soils of Williams (WL). Representative sequences of clones from individual nematodes of particular locations were identified by symbols for the location, the nematode number and unique sequence group it represented. For example, the sequence WL2.1 was from nematode two (2) from Williams (WL) and represented the first unique group (1) of sequences of nematodes from Williams. The overall mean distance between the sequences was 0.013. Sequences of amplicons obtained from the individual nematodes had some degree of genetic variation; both in length and bases and confirmed the mixed sequence profiles from the direct sequencing of the amplicons. There was less sequence divergence in clones of the isolates from Arthur River, Unknown location, Pingelly with respective overall mean distances of 0.001, 0.002, and 0.012, whereas the sequences of nematodes isolated from Katanning and Williams were less identical to each other with overall mean distances of 0.021 and 0.020, respectively. These differences are clearly represented in the phylogenetic tree in which the more similar clones clustered together and those of Williams and Katanning were separated (Fig. 5A).

**Figure 6:**
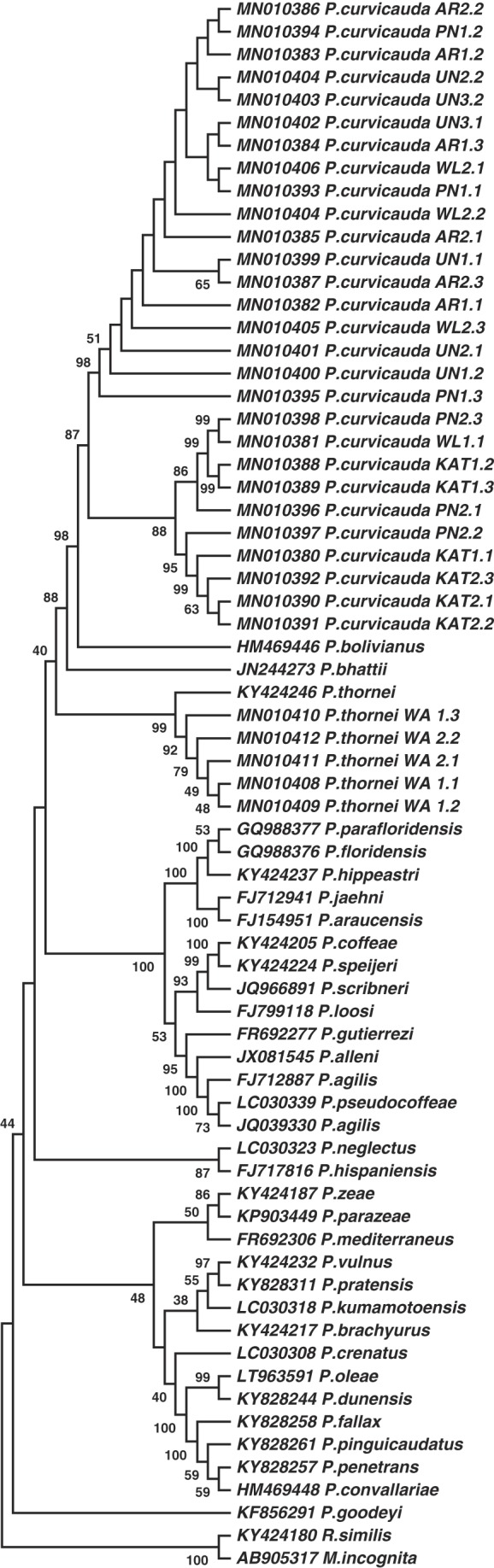
Molecular phylogenetic tree of *P. curvicauda* isolates and sequences representative of 35 *Pratylenchus* species using the partial 18S-ITS1-5.8S-ITS2-28S region of the rDNA. Bootstrap values 30 and above are shown.

**Figure 7:**
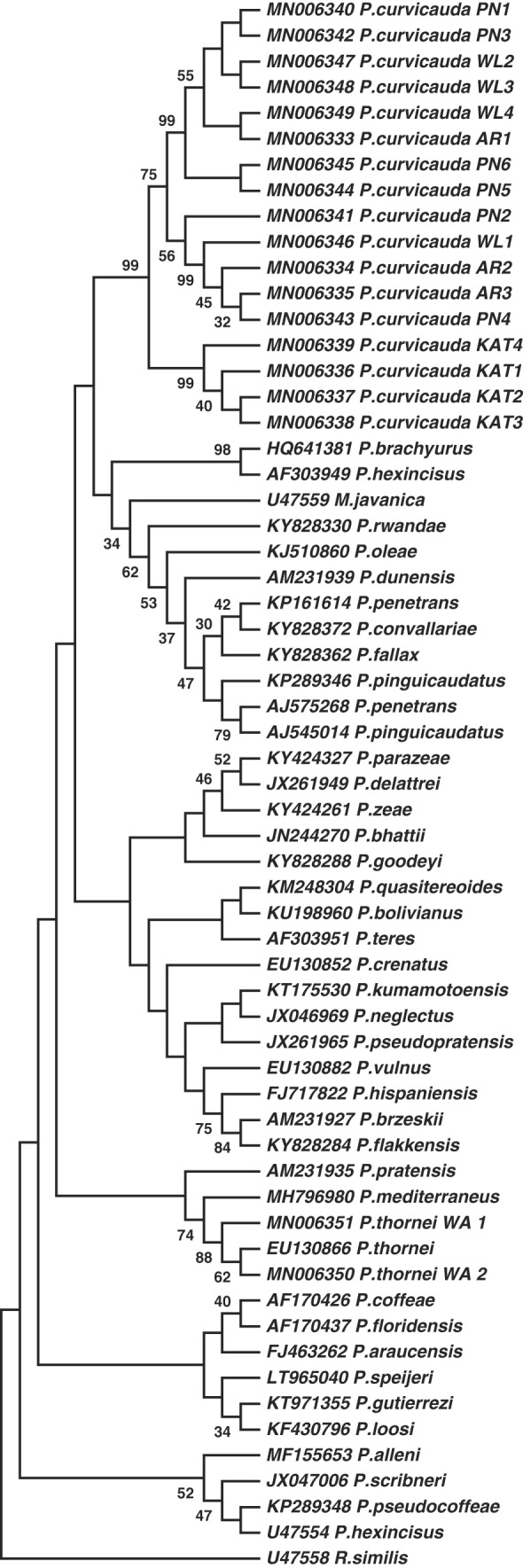
Molecular phylogenetic tree of *P. curvicauda* isolates and sequences representative of 40 *Pratylenchus* species using the 28S-D3 region of the rDNA. Boostrap values 30 and above are shown.

Sequences representing the partial 18S, the ITS1, the 5.8S, the ITS2, and the partial 28S regions of the rDNA were delineated using characterized sequences of other nematodes. The partial 18S (62 nt) and 28S (37 nt) sequences of the amplicons were conserved among the *P. curvicauda* clones except for single nucleotide changes in three positions and a deletion in separate clones in the partial 18S sequences. There was, however, more heterogeneity in the 330 nt long 5.8S region with 24 base changes and 11 positions with insertions/deletions. As was the case for the full sequence analysis, the intra-specific heterogeneity in the ITS1 (311-392 nt) and ITS2 (268-342 nt) regions distinguished the clones from Katanning from those of the four other locations (Fig. 5B,C). Generally, there was more heterogeneity in the ITS2 sequences (overall mean distance of 0.025) than the ITS1 (overall mean distance of 0.010) (Fig. 5B,C).

The *P. curvicauda* sequences were compared to those of similar regions of other *Pratylenchus* nematodes. Redundancy in 919 sequences of the 18S RNA or the partial 18S-ITS1-5.8S-ITS2-partial 28S of *Pratylenchus* nematodes obtained from NCBI was reduced using the CD Hit suite (Huang et al., 2010), with a cut-off of 96% sequence homology, leaving 253 sequences. Of these, 134 were used for phylogenetic analyses and mean distance comparisons with the *P. curvicauda* sequences; this set of sequences had both primers used to amplify the rDNA region of *P. curvicauda* samples so the sequences between the primers were directly compared. The 134 sequences represented 33 recognized and two unspecified *Pratylenchus* species, but there was no sequence for any isolate of *P. curvicauda* as no such sequence has been published or deposited in NCBI or any other sequence database. The representative species included 8 of the 12 reported in Australia. Notably, no sequence of this section of the rDNA used for this analysis for any *Pratylenchus* species originating from Australia was publicly available. So, we amplified a similar region of the rDNA of *P. thornei*, originally cultured from a single nematode and maintained in our laboratory (Nicol et al., 2012), cloned the amplicons, and added sequences of five representative clones (from a total of 10) from two nematodes to the analyses. These sequences, labeled as *P. thornei_WA* were each 826 nt long, relatively shorter than those of *P. curvicauda.* Phylogenetic analyses of the *P. curvicauda* sequences and among the 134 representative sequences of the 35 *Pratylenchus* species explored using the Maximum Likelihood, the Neighbor-Joining and Minimum-evolution approaches of MEGA 6.0 with 1,000 bootstraps indicated *P. curvicauda* formed a highly supported separate clade with *P. bolivianus,* the closest species. In all the molecular phylogenetic trees considered, all the sequences of the same species were grouped together and were clearly distinct from the *P. curvicauda* clones including the *P. thornei_WA* sequences which were in the same clade as the representative *P. thornei* sequence (KY424246.1). Figure 6 is a representation of the phylogenetic tree using representative sequences of 34 of the 35 species, excluding that for the unidentified *Pratylenchus* species. As expected, sequences of the *Pratylenchus* species, including those of *P. curvicauda* were clearly distinct from representative sequences of the two outgroups of the genera *Meloidogyne* and *Radopholus.*

The distinct grouping of the *Pratylenchus* species was supported by the intra-species mean distances in sequences of the species analyzed. The overall mean distance of the 134 *Pratylenchus* sequences with the 28 of *P. curvicauda* was 0.202, and was higher than the overall intra-species mean distances for sequences of each of the individual species. Notably, the mean distances for representative sequences of eight *Pratylenchus* species commonly found in Australia were mostly higher than that for the *P. curvicauda* clones (0.013): *P. neglectus* (0.051), *P. penetrans* (0.043), *P. vulnus* (0.051), *P. brachyurus* (0.059), *P. coffeae* (0.036), *P. crenatus* (0.068), *P. goodeyi* (0.311), and *P. zeae* (0.069).

### Comparison of 28S-D3 sequence of *P. curvicauda* with those of other *Pratylenchus* spp.

Sequences of the 28S-D3 regions of the rDNA were also analyzed; the aim was to characterize and distinguish the *P. curvicauda* further from other *Pratylenchus* species particularly those present in Australia for which similar sequences were available. The 17 consensus sequences of clones of the amplicons from the 28S-D3 regions of *P. curvicauda* analyzed were between 314 and 318 nt long with an overall mean diversity of 0.085. The sequences of nematodes from Katanning were less diverse with an overall mean diversity of 0.003; those from Pingelly, Arthur River and Williams had higher overall mean diversities, respectively, 0.061, 0.059, and 0.039. Sequences from the Western Australia *P. thornei* isolate had a mean diversity of 0.014.

From 842 sequences at the NBCI databases for the 28S-D3 region of the rDNA of *Pratylenchus* species, a set of 118 was used to study relationships (i.e. to construct phylogenetic trees and estimate mean distances) after reducing redundancy (with a cut-off of 98% similarity) and delineating the sequences based on those available for isolates of *P. quasitereoides* (215 bp); the latter exercise resulted in the use of less than the full sequence of the amplicons of *P. curvicauda* in the phylogenetic analyses. The resulting sequences represented 39 recognized and 12 unspecified *Pratylenchus* species including sequences of Australian isolates; 10 for *P. quasitereoides* (Hodda et al., 2014) and 2 for *P. thornei* (Subbotin et al., 2008). All molecular phylogenetic trees constructed using Maximum Likelihood, the Neighbor-Joining and Minimum-evolution approaches of MEGA 7.0 with 1,000 bootstraps grouped *P. curvicauda* as a separate *Pratylenchus* species, distinct from all the other *Pratylenchus* species and the outgroups, *Meloidogyne javanica*, and *R. similis*. Figure 7 shows the relationship of the *P. curvicauda* sequences with representative sequences of 40 species of *Pratylenchus* including a representative sequence of the 10 available for *P. quasitereoides*. Notably, because of the conservation of the 28S-D3 sequences even between species of different genera, the *M. javanica* used appears to be more closely-related to some of the *Pratylenchus* spp. The overall mean distance of all the sequences of the *Pratylenchus* species including those of *P. curvicauda* and *P. thornei*_*WA* was 0.167 compared to 0.12 among the 17 sequences of the *P. curvicauda* alone. Contrary to features of the partial 18S-ITS1-5.8S-ITS2-partial 28S, the overall mean distances among both the full sequences of amplicons of the 28S-D3 of *P. curvicauda* and the portions used for the phylogenetic analyses, respectively, 0.085 and 0.12, were higher than the intra-species value for the *Pratylenchus* spp. with multiple sequences in the data set used. There was a relatively lower diversity in the 28S-D3 sequences than the partial 18S-ITS1-5.8S-ITS2-partial 28S of species commonly found in Australia, namely, *P. neglectus* (0.013), *P. vulnus* (0.009), *P. brachyurus* (0.025), *P. crenatus* (0.024), and *P. zeae* (0.055) except for *P. penetrans* (0.058), *P. coffeae* (0.055), and *P. thornei* (0.019) where the opposite was true. Also, the separate phylo-grouping of *P. curvicauda* from the Australian isolates of *P. quasitereoides* (overall mean distance of 0.023) is clearly supported by the differences in the diversity in the representative sequences of the two species.

## Discussion

This study is the first to describe and characterize *P. curvicauda* isolated from the grainbelt of Western Australia. The type nematodes were serendipitously identified from mixed infestations in soils from four locations; Pingelly, Arthur River, Williams and Katanning, during a study initially on the incidence of *P. quasitereoides.* The existence of morphotypes among the *Pratylenchus* species was obvious during the assessment as a number of the morphometric and morphological features of the *P. curvicauda* overlapped with those of other species. However, using published morphometric and morphological data and comparison with *in vitro*-maintained *P. thornei* population developed from a single female, we showed that the species of *Pratylenchus* we isolated from the wheat and barley fields was conspecific to *P. curvicauda*. In fact, for most of the field samples studied, a *Pratylenchus* species corresponding to the published description of *P. quasiterioides* (Hodda et al., 2014) was not found. This report may now complicate the management of root lesion nematodes in the Western Australia grainbelt. There has been no follow-up study on *P. curvicauda* in Australia since it was first identified in *Trifolium* spp. in metropolitan Perth, Western Australia, so, the findings of this study now make it easier to identify *P. curvicauda* in soils, and to distinguish it from other root lesion nematodes, providing an important first step to developing effective management strategies for this nematode.

Sequences of the partial 18S-ITS1-5.8S-ITS2-partial 28S and/or the 28S-D3 regions of the rDNA of over 1,000 isolates of ca 45 *Pratylenchus* species isolated from around the world are currently available in public databases. Some of these have been used separately or in combination with traditional methods to accurately characterize and to fingerprint *Pratylenchus* species (Waeyenberge et al., 2000; Waeyenberge et al., 2009; De Luca et al., 2011). The use of the former approach has been very successful because of the inter-genetic variation in the ITS1 and ITS2 sequences of different species, notwithstanding any intra-genetic variations in individual nematodes of the same species (Duncan et al., 1999; Waeyenberge et al., 2000; Waeyenberge et al., 2009; Wang et al., 2015). During the analyses of our data, it was surprising to note that although there are about a dozen *Pratylenchus* species of economic importance reported in Australia, no public molecular data, especially of the partial 18S-ITS1-5.8S-ITS2-partial 28S region of the rDNA, was available for any isolate of *Pratylenchus* spp. of Australian origin. Sequences of the 28S-D3 regions of two isolates of *P. thornei* (EU130866.1 and EU130869.1) from Queensland and South Australia (Subbotin et al., 2008) and those from the recently re-described *P. quasitereoides* (Hodda et al., 2014) were the only publicly available nucleotide sequences of *Pratylenchus* species besides the transcriptomes of *P. thornei* and *P. zeae* (Nicol et al., 2012; Fosu-Nyarko et al., 2015). Our sequencing of these rDNA regions for *P. curvicauda* and *P. thornei-WA* therefore provides the first sequences of the partial 18S-ITS1-5.8S-ITS2-partial 28S regions of the rDNA for any *Pratylenchus* spp. of Australian origin. These, and those of the 28S-D3 sequences of *P. curvicuada* and *P. thornei-WA* together with those already available provide a good lead to using molecular data to accurately diagnose *Pratylenchus* spp. in Australia for better and specific management of their damage or infestations.

By applying sequences of the rDNA, we clearly established that the isolates of the *P. curvicauda* we analyzed were of the same species and were distinct from other *Pratylenchus* species including economically important pest species such as *P. neglectus, P. penetrans*, *P. thornei*, *P. zeae*, and *P. quasitereoides* commonly reported in Western Australia. The lower and significantly different overall mean distances in the partial 18S-ITS1-5.8S-ITS2-partial 28S sequences of the 80 clones generated from 14 individual *P. curvicauda* adult females compared to those of other *Pratylenchus* species have two implications. First, that the molecular data validated the traditional methods used for concluding the isolates of *P. curvicauda* from soils at Pingelly, Katanning, Arthur River and Williams were of the same *Pratylenchus* species. Second, the lower discrete diversity in the sequences of the isolates implies they could not be conspecific with any other *Pratylenchus* species compared, including the 11 economically important species reported in Australia. Similar conclusions could be drawn from the analyses of sequences of the 28S-D3 expansion regions where again, the measure of diversity, the overall mean distance for sequences of both the full 28S-D3 amplicons of the isolates and that for the portions used for phylogenetic analyses were both significantly different from those within sequences of isolates of each of the 39 *Pratylenchus* species studied. The results further affirm the distinct speciation of the *P. curvicauda*. In the intra-species sequence analyses of the *P. curvicauda* isolates from the four locations and the inter-species phylogenetic analyses with the partial 18S-ITS1-5.8S-ITS2-partial 28S, and the 28S-D3 sequences, and the overall mean distances between the species, it was consistently evident that the isolates from Katanning formed a small clade whereas isolates from the other three locations were randomly distributed to groups in separate clades. While it is difficult to assess which of the locations the nematodes may have first been introduced or may have originated, it cannot be ruled out that the species may have spread over the grainbelt region during the long period of cropping in these areas.

Direct phylogenetic relationship could not be established with all Australian isolates of *Pratylenchus* species because sequences of the rDNA of such isolates were not available except for *P. quasitereoides* (previously *P. teres*) and *P. thornei* and, no pure cultures of any of the isolates was available. However, it is plausible that because the consensus rDNA sequences of the *Pratylenchus* species used for this study were representative of each taxon based on the distinct diversities, the *P. curvicauda* could not be conspecific with any of the other *Pratylenchus* species in Australia, namely, *P. neglectus, P. vulnus, P. brachyurus, P. crenatus*, *P. thornei*, *P. zeae, P. penetrans, P. coffeae, P. goodeyi*, and *P. thornei.* The only *Pratylenchus* species reported in Australia for which we could not compare morphological features, morphometric data or molecular sequence information directly was *P. jordanensis*; the adoption of this species as a junior synonym of *P. scribneri* or *P. zeae* (Castillo and Vovlas, 2007) and as such the unavailability of specific data indicates further comparison was not necessary and that by deduction the *P. curvicauda* identified in this study could not be conspecific to *P. jordanensis*.

The pest status of *P. curvicauda* in Australia is currently unknown; its isolation from the grainbelt of Western Australia makes it more compelling for studies into its incidence and prevalence to be undertaken. In general, an investigation into its biology, particularly its life cycle and host range, are necessary so that effective strategies can be developed to manage any damage they may cause to important crops such as wheat and barley, which are the major crops grown in the farming areas where the nematodes were identified. Also, information on the nematode’s interaction with hosts would aid the identification of resistance genes or loci for the development of tolerant or resistant cultivars, as one of the important components for managing these belowground pests of economic importance (Jones et al., 2016).
